# Prognostic value of cardiovascular magnetic resonance in immune checkpoint inhibitor‐associated myocarditis: A systematic review and meta‐analysis

**DOI:** 10.1002/cai2.109

**Published:** 2024-04-15

**Authors:** Wenhua Song, Nan Zhang, Tonglian Lv, Yang Zhao, Guangping Li, Gary Tse, Tong Liu

**Affiliations:** ^1^ Tianjin Key Laboratory of Ionic‐Molecular Function of Cardiovascular Disease, Department of Cardiology, Tianjin Institute of Cardiology Second Hospital of Tianjin Medical University Tianjin China; ^2^ Department of Radiology Second Hospital of Tianjin Medical University Tianjin China; ^3^ School of Nursing and Health Studies Hong Kong Metropolitan University Hong Kong China; ^4^ Cardiac Electrophysiology Unit Cardiovascular Analytics Group, PowerHealth Limited Hong Kong China

**Keywords:** cardiovascular magnetic resonance, immune checkpoint inhibitors, myocarditis

## Abstract

**Background:**

Immune checkpoint inhibitors (ICI) are increasingly used in the first‐line treatment of malignant tumors. There is increasing recognition of their cardiotoxicity and, in particular, their potential to lead to myocarditis. Cardiovascular magnetic resonance (CMR) can quantify pathological changes, such as myocardial edema and fibrosis. The purpose of this systematic review and meta‐analysis was to examine the evidence for the roles of CMR in predicting prognosis in ICI‐associated myocarditis.

**Methods:**

PubMed, Cochrane Library, and Web of Science databases were searched until October 2023 for published works investigating the relationship between CMR parameters and adverse events in patients with ICI‐associated myocarditis. The analysis included studies reporting the incidence of late gadolinium enhancement (LGE), T1 values, T2 values, and CMR‐derived left ventricular ejection fraction (LVEF). Odds ratios (OR) and weighted mean differences (WMD) were combined for binary and continuous data, respectively. Newcastle‐Ottawa Scale was used to assess the methodological quality of the included studies.

**Results:**

Five cohort studies were included (average age 65–68 years; 25.4% female). Of these, four studies were included in the meta‐analysis of LGE‐related findings. Patients with major adverse cardiovascular events (MACE) had a higher incidence of LGE compared with patients without MACE (OR = 4.18, 95% CI: 1.72–10.19, *p* = 0.002). A meta‐analysis, incorporating data from two studies, showed that patients who developed MACE exhibited significantly higher T1 value (WMD = 36.16 ms, 95% CI: 21.43–50.89, *p* < 0.001) and lower LVEF (WMD = − 8.00%, 95% CI: −13.60 to −2.40, *p* = 0.005). Notably, T2 value (WMD = −0.23 ms, 95% CI: −1.86 to −1.39, *p* = 0.779) was not associated with MACE in patients with ICI‐related myocarditis.

**Conclusions:**

LGE, T1 value, and LVEF measured by CMR imaging have potential prognostic value for long‐term adverse events in patients with ICI‐related myocarditis.

## Graphical abstract

Patients with major adverse cardiovascular events (MACE) showed higher late gadolinium enhancement frequency (OR = 4.18, 95% CI: 1.72–10.19, *p* = 0.002), higher T1 value (WMD = 36.16 ms, 95% CI: 21.43–50.89, *p* < 0.001), and lower left ventricular ejection fraction (WMD: −8.00%, 95% CI: −13.60 to −2.40, *p* = 0.005) compared with patients without MACE.

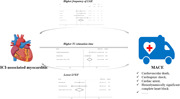

**Abbreviations**
CMR: cardiovascular magnetic resonance
ICI: immune checkpoint inhibitors
LGE: late gadolinium enhancement
LVEF: left ventricular ejection fraction
MACE: major adverse cardiovascular events
OR: odds ratios
WMD: weighted mean difference

## 1 INTRODUCTION

The development of immune checkpoint inhibitors (ICI) represents one of the great advances in the field of malignant tumor treatment in recent years [1, 2, 3]. ICIs are monoclonal antibodies that work by blocking the interaction between tumor cells and immune cells expressing immune checkpoint molecules, thereby promoting the immune system to attack and kill cancer cells [4, 5]. However, nonspecific activation of the immune system can lead to a series of immune‐related adverse events involving multiple organs [6, 7, 8, 9]. Myocarditis due to ICI is rare, with an incidence of approximately 0.1% [10]. With the rapid increase in the number of related cases, it is suggested that the true incidence of ICI‐related myocarditis may be underestimated [11]. ICI‐related myocarditis has a mortality rate of 30%–50% [12] and is classified as a serious adverse event [13, 14]. Endocardial biopsy is the gold standard for diagnosis but is limited by its trauma and potential complications [15].

Cardiovascular magnetic resonance (CMR) is a noninvasive imaging method that can be used to assess cardiac dysfunction and damage in patients with suspected myocarditis [16, 17]. T1 and T2 mapping can quantitatively measure the prolonged longitudinal relaxation time in myocarditis, reflecting the dynamic pathological changes of myocarditis, from edema to necrosis and fibrosis of cardiac tissue. This article searches published literature to analyze CMR parameters in patients with ICI‐related myocarditis who have major adverse cardiovascular events (MACE) and summarizes the value of CMR in the prognosis of ICI‐related myocarditis.

## 2 METHODS

### 2.1 Search strategy

In this meta‐analysis, we searched PubMed, Cochrane Library, and Web of Science to identify all eligible studies exploring the value of CMR in the assessment of adverse events in patients with ICI‐related myocarditis. The keywords used were “immune checkpoint inhibitor” and “cardiovascular magnetic resonance.” Only studies with human subjects were included. The search ended in October 2023.

### 2.2 Selection criteria

The inclusion criteria were all clinical studies analyzing the relationship between CMR parameters and long‐term adverse events in patients with ICI‐related myocarditis. Patients with a definite diagnosis of ICI‐related myocarditis were included in the study, and all participants received ICI therapy. The outcomes should be available in the literature. However, those having only abstracts but no full text and did not report the occurrence of MACE were excluded. Case reports, conference summaries, guidelines, expert consensus, reviews, non‐English reports, and animal experiments were also excluded. There were no restrictions on the type of study design.

### 2.3 Data extraction and quality assessment

Literature search, study selection, and data extraction were performed independently by two reviewers (W. S. and N. Z.). Disagreements were resolved by consensus. For each study, we recorded CMR parameters and long‐term adverse events in patients with ICI‐related myocarditis. Datas including authors, year of publication, study design, sample size, sex, and age of subjects, and CMR parameters, such as late gadolinium enhancement (LGE), T1 value, T2 value, and left ventricular ejection fraction (LVEF), as well as follow‐up duration, frequency, and definition of MACE events were extracted. Of these, all CMR studies by Thavendiranathan et al. were performed on 1.5‐T or 3‐T machines. For consistency with the paper by Zhao et al., only T1 and T2 values obtained from the 1.5‐T machine were included. Numerical data as presented in the article was used. We also evaluated these data based on survival curves that were not reported in some studies. The Newcastle‐Ottawa Scale (NOS) was used to evaluate the methodological quality of included studies. The NOS consists of eight items organized into three dimensions, including selection, comparability, and outcome (cohort study) or exposure (case‐control study) depending on the type of study. For each item, a range of response options were provided. A point system was used to provide a semiquantitative assessment of research quality, with the highest quality studies receiving a maximum of one point per item, except for items related to comparability, which are allowed to be assigned two points. NOS ranges from zero to nine points [18].

### 2.4 Data analysis

Odds ratios (OR) and 95% confidence interval (CI) were reported for binary data, and weighted mean differences (WMD) were used for continuous data. The inconsistency index test and *Q* test were used to assess heterogeneity between studies. *I* ^2^ > 50% and *Q*‐test *p* < 0.1 indicated statistically significant heterogeneity. Fixed‐effects models were used when the degree of statistical heterogeneity was low, otherwise random‐effects models were used for meta‐analysis. A sensitivity analysis was performed by excluding low‐quality studies to determine whether the results remained robust. Begg's test and Egger's test were used to test the bias of the included study. The original studies reported LGE rates and therefore ORs were used to summarize effect estimates in the meta‐analysis, whereas parameters, such as T1 and T2 value and LVEF, were provided in the original literature as continuous variables, and therefore WMDs were reported in the meta‐analysis. All tests were performed using Stata (Version: SE 12.0).

## 3 RESULTS

**Figure 1:**
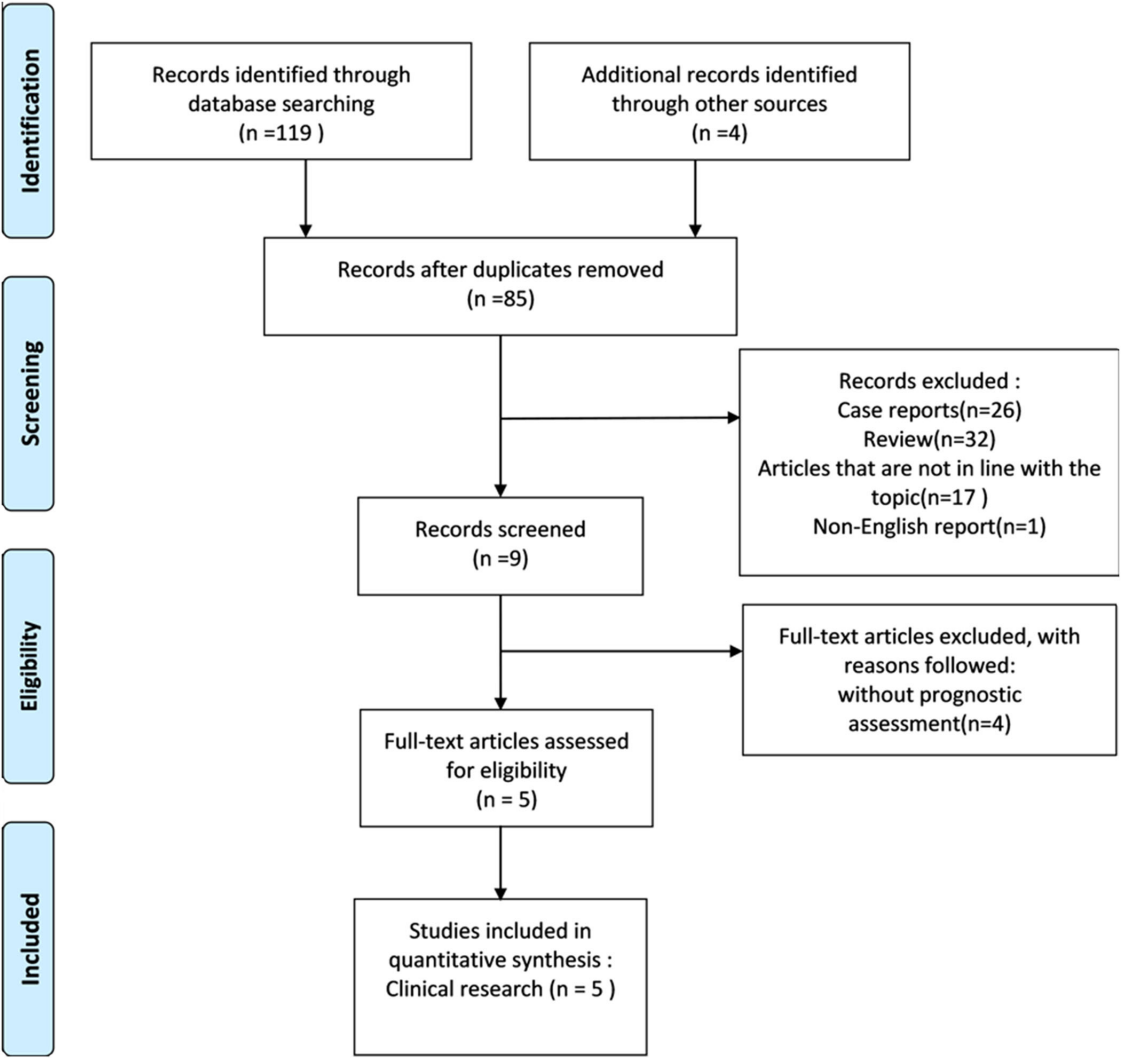
Flowchart of the study selection process.

The initial search yielded 119 results and an additional four articles were identified from the references. First, a total of 38 articles were excluded through title/abstract screening and deletion of duplicate literature and secondary literature. Eighty‐five studies were selected and read in full and irrelevant literature, reviews, case reports, non‐English reports, and other literature were eliminated. Five articles were finally included [4, 19, 20, 21, 22], three of which were retrospective studies [19, 20, 22], one was a prospective study [4], and one was a two‐way study [21]. A flowchart detailing the search process, study identification, and inclusion and exclusion is shown in Figure 1.

A total of 359 subjects from five studies were analyzed. The average age of these five studies ranged from 65 to 68 years. With the exception of one study, which did not report the sex ratio; [20] females comprised 25.4% (78/307) of all patients in the remaining four studies. All patients in five studies received ICI therapy and were diagnosed with ICI‐related myocarditis. The diagnosis was based on one of the following: (1) Standard features demonstrated by histopathology or (2) Diagnostic criteria for clinically suspected myocarditis based on European Society of Cardiology (ESC) guidelines [15]. The study's follow‐up period was 3–6 months. All patients enrolled in the study underwent CMR and reported their results, including LGE, T1 and T2 value, and LVEF. The basic characteristics of the included studies are shown in Table 1.

**Table 1:** Characteristics of included studies.

Reference	Study region	Study design	No. (M,F)	Age (years)	ICI	CMR equipment	LGE	T1 value	T2 value	LVEF	Diagnosis of ICI‐M	Median follow‐up time (days)	No. of MACE	MACE	Newcastle‐Ottawa Scale
Mahmood et al.^21^	An eight‐center institutional registry	Retrospective and prospective	35 (25,10)	65 ± 13	Pembrolizumab; Nivolumab; Ipilimumab; Tremelimumab; Atezolizumab; Avelumab; Durvalumab	NA	+	−	−	+	Histological features ESC guidelines	102	16	A composite of cardiovascular death, cardiogenic shock, cardiac arrest, and hemodynamically significant complete heart block	6
Zhang et al.^4^	USA, Canada, and Europe	Prospective	103 (73,30)	65.6 ± 15.3	Nivolumab; pembrolizumab; ipilimumab; tremelimumab; avelumab; atezolizumab	1.5 or 3 T CMR system	+	−	−	NA	Histological features ESC guidelines	148.5	41	A composite of cardiovascular death, cardiac arrest, cardiogenic shock, and complete heart block (CHB) requiring a pacemaker.	7
Thavendiranathan et al.^19^	An international multicenter registry	Retrospective	136 (108,28)	66.3 ± 13.1	Anti‐CTLA4; anti‐PD1; anti‐PDL1	1.5‐T or 3‐T Siemens (Siemens, Erlangen, Germany); 1.5‐T Philips(Philips, Best, the Netherlands)	−	+	+	−	Histological features ESC guidelines	158	27	A composite of cardiovascular death, cardiac arrest, cardiogenic shock, and complete heart block requiring a pacemaker	6
Cadour et al.^22^	France	Retrospective	33 (23,10)	68 ± 14	NA	1.5‐T Siemens (Magnetom Amira, Avanto, or Aera); 3.0‐T Siemens (Magnetom Skyra); 1.5‐T Philips (Ingenia) scanner	+	−	−	−	EMB Bonaca criteria	92	21	A composite of death from cardiovascular causes (including sudden death), documented sustained (>30 s) ventricular tachycardia, ventricular fibrillation, complete atrioventricular heart block, and cardiogenic shock	5
Zhao et al.^20^	China	Retrospective	52 (NA)	NA	Anti‐CTLA4; anti‐PD1; anti‐PDL1	1.5‐T scanner (Magnetom Aera, Siemens Healthcare)	−	+	+	+	ESC guidelines	171	14	A composite of cardiovascular death, cardiac arrest, cardiogenic shock, and complete heart block	7

Abbreviations: anti‐CTLA4, anti‐cytotoxic T‐lymphocyte associated protein 4; anti‐PD1, anti‐programmed cell death protein 1; anti‐PDL1, anti‐programmed death‐ligand 1; NA, not available.

This study included five clinical studies to evaluate the reference value of CMR parameters for the occurrence of long‐term adverse events in patients with ICI‐related myocarditis. For the analysis of T1 mapping and T2 mapping, only two studies reported relevant data, and we conducted a meta‐analysis of all available data to better understand the significance of CMR in determining prognostic information in patients with ICI‐related myocarditis.

Regarding the definition of MACE, the five included studies had similar definitions: in four studies this was defined as cardiovascular death, cardiogenic shock, cardiac arrest, and hemodynamically significant complete heart block [4, 19, 20, 21], whereas the remaining study defined it as death from cardiovascular causes (including sudden death), documented sustained (>30 s) ventricular tachycardia, ventricular fibrillation, complete atrioventricular heart block, and cardiogenic shock [22]. The pooled incidence of MACE among all patients was 33.1% (119/359).

### 3.1 LGE and MACE

**Figure 2:**
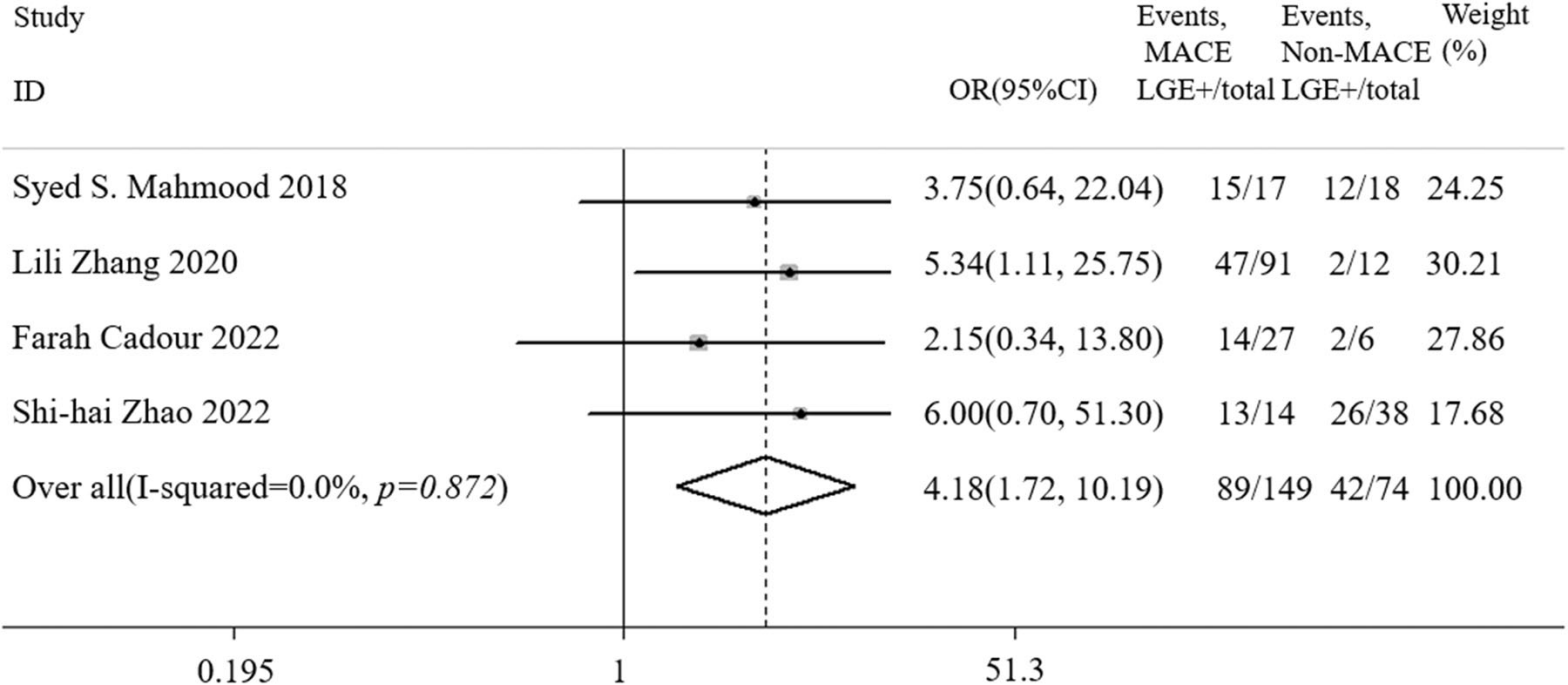
Forest plot comparing the clinical outcomes in patients with and without LGE.

**Figure 3:**
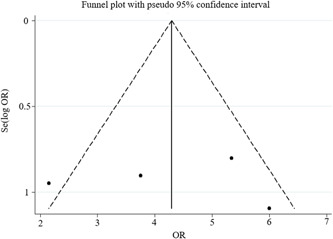
Funnel chart of meta‐analysis. OR, odds ratio; Se, standard error.

**Figure 4:**
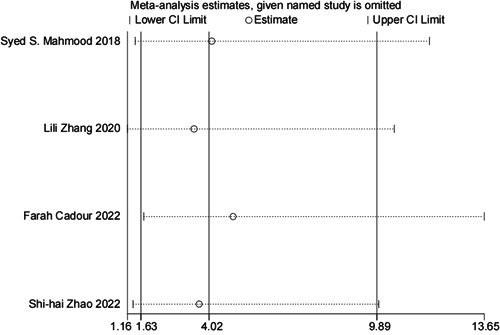
Sensitivity analysis of meta‐analysis.

The work of Thavendiranathan et al [19]. was not included in the meta‐analysis in this section because they did not provide LGE‐related data. The four studies providing LGE included a total of 223 participants. In the meta‐analysis of the remaining four studies, the heterogeneity between studies was not statistically significant (*I* ^2^ = 0.00%, *p* = 0.874), so the fixed‐effects model was used for meta‐analysis. The meta‐analysis results showed that compared with patients without MACE, patients who developed MACE had higher odds of developing LGE (OR = 4.18, 95% CI: 1.72–10.19; *p* = 0.002; Figure 2). Sensitivity analysis of four studies indicated that the results of this meta‐analysis are robust and reliable. Begg's test and Egger's test found that the meta‐analysis did not show significant publication bias (*p* = 1.000 and *p* = 0.996, respectively) (see Figures 3 and 4).

### 3.2 T1 and T2 value, LVEF, and MACE

**Figure 5:**
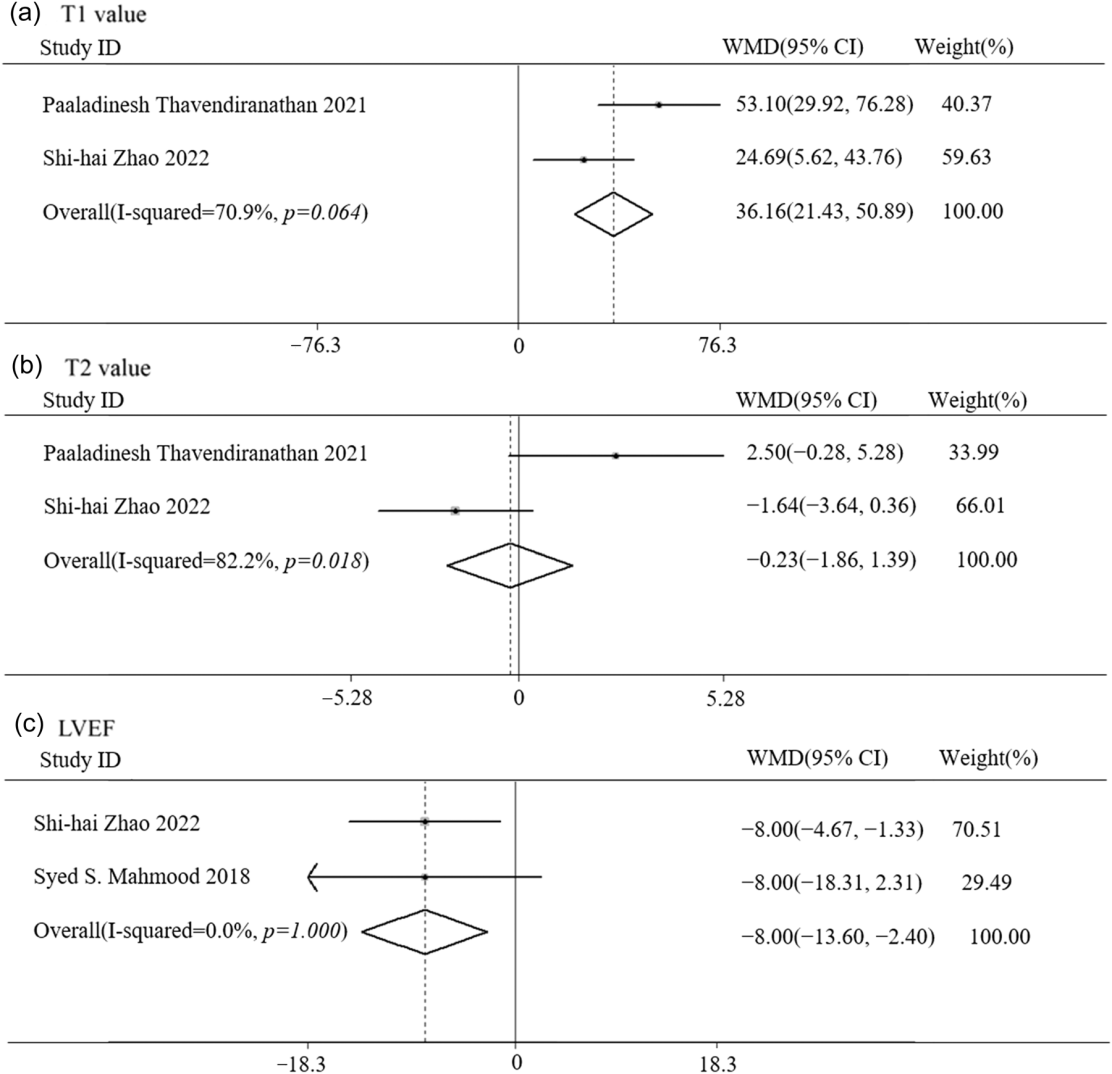
Forest plot comparing the clinical outcomes by (a) T1 value, (b) T2 value, and (c) LVEF. LVEF, left ventricular ejection fraction; WMD, weighted mean difference.

Only two of the included studies reported data on T1 and T2 value and LVEF, so the numbers of these studies included in the meta‐analysis were 119, 111, and 97 respectively [19, 20]. For LVEF, there was no significant heterogeneity between the two studies (*I* ^2^ = 0.00%, *p* = 1.000), so a fixed‐effects model was used. In contrast, for T1 and T2 value, there was significant heterogeneity between the two studies (T1 value: *I* ^2^ = 70.90%, *p* = 0.064; T2 value: *I* ^2^ = 82.2%, *p* = 0.018), so for this meta‐analysis, a random‐effects model was used. Patients who developed MACE showed significantly higher T1 value (WMD = 36.16 ms, 95% CI: 21.43–50.89, *p* < 0.001) and lower LVEF (WMD = −8.00%, 95% CI: −13.60 to −2.40, *p* = 0.005). However, T2 value showed no correlation (WMD = −0.23 ms, 95% CI: −1.86 to −1.39, *p* = 0.779, Figure 5).

## 4 DISCUSSION

As the importance of ICI‐related myocarditis is increasingly recognized [23, 24, 25, 26], international guidelines have provided recommendations on its diagnosis and treatment [10]. CMR plays a key role in exploring ICI‐related myocarditis in clinically suspected cases. This meta‐analysis aims to amalgamate insights from published studies, yielding comprehensive estimates regarding the predictive role of CMR in adverse events for patients with ICI‐related myocarditis. We found that LGE, high T1 values, and low LVEF on CMR were associated with MACE in patients with ICI‐related myocarditis, whereas T2 values were not.

In LGE, diseased areas show an accumulation of gadolinium concentration, resulting in brighter areas. In myocarditis, nonischemic LGE is common and is related to myocardial edema, fibrosis, and other injuries, and has a poor clinical prognosis. This study found that the proportion of LGE in MACE patients was higher, which is consistent with the above conclusion. In addition, myocardial fibrosis or scarring reflected by LGE may be subacute or chronic sequelae of myocardial inflammation, so the formation and accumulation of myocardial fibrosis may take some time to develop on CMR or biopsy to determine the final degree of cardiac involvement in myocarditis. In a rat model of autoimmune myocarditis, approximately 20% of rat hearts developed LGE 2 weeks after immunization, rising to 62.5% after 5 weeks [27]. However, since most of the studies included in this article were retrospective reports, the timing of the CMR examination was determined by the clinician. This may be influenced by clinical symptom severity and technical availability, and the exact timing of patients receiving CMR examinations is unknown. Prospective clinical studies are needed to standardize and further elucidate the relationship between LGE and time in patients with ICI‐related myocarditis.

CMR with T1 and T2 mapping has higher sensitivity in detecting inflammation, edema, and fibrosis. According to the Lake Louis Criteria (LLC) updated in 2018, the main diagnostic criteria for myocarditis are: (1) evidence of nonischemic myocardial injury (abnormal T1, ECV, or LGE); (2) evidence of myocardial edema (prolonged T2) [28]. Compared with other noninvasive imaging techniques, T1 and T2 mapping are one of the main reference methods for the diagnosis and prognosis of patients with myocarditis. In acute myocarditis, the elevated T1 and T2 values reflect inflammation and edema. In addition, T1 mapping can identify fibrosis in the early stages of myocarditis [29, 30]. The results of our meta‐analysis showed that patients with MCAE events tended to show increased T1 values but not T2 values. The possible reasons are as follows: First, most of the data for parametric mapping imaging involved in this study, except for LGE, came from the study of Thavendiranathan et al.,[19] in which the increase in T1 value was consistent with histopathological inflammatory cell infiltration, while no obvious abnormality was found in T2 value. The authors suggested that the difference in detected abnormal values between T1 and T2 value may reflect the low sensitivity of T2 mapping sequences in detecting myocarditis. Second, the research team pointed out that the high incidence of abnormal T1 value reflects the greater degree of myocardial damage caused by myocarditis, the presence of early myocardial fibrosis, and even persistent indolent myocardial inflammation [19]. Therefore, in patients with acute ICI‐related myocarditis, prolonged T1 versus T2 value are more likely to be identified. Furthermore, one potential reason for the lack of an independent relationship between T2 value and MACE is that T2 may reflect reversible edema, which may have less impact on prognosis. Third, elevated T1 values also reflect underlying cardiomyopathy and other cardiovascular risk factors in the past, which may affect the prognosis of cancer treatment [19]. Regarding LVEF, meta‐analysis shows that MACE patients have lower LVEF, suggesting that their cardiac function was relatively poor and was related to poor clinical prognosis.

Several limitations of this study should be recognized. First, this meta‐analysis used summary data reported in published articles rather than individual participant‐level data. Second, because most studies are retrospective reports, many clinical data are unavailable, such as the area of fibrosis, the degree of myocardial edema, and the time from diagnosis of ICI‐related myocarditis to CMR examination, so the analysis of the results is limited. Third, for the analysis of T1 and T2 values and LVEF, the number of included cases was small, and different machines (Siemens and Philips) were used for T1/T2 mapping. For the above parameters, only two studies provided specific data, which may cause bias, and the clinical significance will be further updated as relevant studies are published in the future. Fourth, the clinical studies included in this article come from multiple countries and regions, and the investigation periods of the included studies span more than 4 years, so there may be differences in technology, diagnosis, and treatment. According to NOS, the low scores of some studies were also one of the limitations of this study. In the future, more large‐scale and high‐quality studies are needed to clarify the clinical significance of CMR on the prognosis of ICI myocarditis. Finally, only five studies were identified and included in this meta‐analysis, yielding a relatively small sample size. Therefore, prospective studies with larger sample sizes, standardization of imaging acquisition times, and application of LGE protocols are needed to further elucidate the pathophysiology of ICI‐related myocarditis.

## 5 CONCLUSION

CMR manifestations of ICI‐related myocarditis included myocardial dysfunction, edema, and fibrosis. Comprehensive CMR examination, especially LGE, T1 value, and LVEF have potential prognostic value and help guide clinical treatment.

### 5.1 Clinical perspectives

#### 5.1.1 Clinical competencies

CMR can quantify pathological changes, such as myocardial edema and fibrosis. It plays an important role in early identification, monitoring, and prognostic assessment of ICI‐related myocarditis.

#### 5.1.2 Translational outlook

LGE, T1 value, and LVEF on CMR imaging may predict prognosis in ICI‐related myocarditis.

## AUTHOR CONTRIBUTIONS

**Wenhua Song**: Data curation (equal); Methodology (equal); Writing—original draft (equal). **Nan Zhang**: Methodology (equal); Validation (equal). **Tonglian Lv**: Investigation (equal); Methodology (equal); Writing—original draft (equal). **Yang Zhao**: Writing—review & editing (equal). **Guangping Li**: Project administration (equal); Supervision (equal). **Gary Tse**: Supervision (equal); Writing—review & editing (equal). **Tong Liu**: Funding acquisition (equal); Supervision (equal); Writing—review & editing (equal).

## CONFLICT OF INTEREST STATEMENT

Professor Tong Liu is a member of the *Cancer Innovation* Editorial Board. To minimize bias, he was excluded from all editorial decision‐making related to the acceptance of this article for publication. The remaining authors declare no conflict of interest.

## ETHICS STATEMENT

Not applicable.

## INFORMED CONSENT

Not applicable.

## Data Availability

Data sharing is not applicable to this article as no new data were created or analyzed in this study.
